# Respective influence of vertical mountain differentiation on debris flow occurrence in the Upper Min River, China

**DOI:** 10.1038/s41598-020-68590-2

**Published:** 2020-07-16

**Authors:** Mingtao Ding, Tao Huang, Hao Zheng, Guohui Yang

**Affiliations:** Faculty of Geosciences and Environmental Engineering, Southwest Jiaotong University, Chengdu, 611756 China

**Keywords:** Environmental sciences, Natural hazards

## Abstract

The generation, formation, and development of debris flow are closely related to the vertical climate, vegetation, soil, lithology and topography of the mountain area. Taking in the upper reaches of Min River (the Upper Min River) as the study area, combined with GIS and RS technology, the Geo-detector (GEO) method was used to quantitatively analyze the respective influence of 9 factors on debris flow occurrence. We identify from a list of 5 variables that explain 53.92%% of the total variance. Maximum daily rainfall and slope are recognized as the primary driver (39.56%) of the spatiotemporal variability of debris flow activity. Interaction detector indicates that the interaction between the vertical differentiation factors of the mountainous areas in the study area is nonlinear enhancement. Risk detector shows that the debris flow accumulation area and propagation area in the Upper Min River are mainly distributed in the arid valleys of subtropical and warm temperate zones. The study results of this paper will enrich the scientific basis of prevention and reduction of debris flow hazards.

## Introduction

Debris flows are a common type of geological disaster in mountainous areas^1,2^, which often causes huge casualties and property losses^3,4^. To scientifically deal with debris flow disasters, a lot of research has been carried out from the aspects of debris flow physics^5–9^, risk assessment^10–12^, social vulnerability/resilience^13–15^, etc. Jointly influenced by unfavorable conditions and factors for social and economic development, the Upper Min River is a geographically uplifted but economically depressed region in Southwest Sichuan. Being prone to mountain hazards such as landslides and debris flows^16^. This region has been ecologically fragile with constant disturbance by human activities and under the chain effect of secondary mountain hazards induced by the Grand Earthquake.

According to the research on the typical debris flow disaster events in the upper Min River^17–22^, it is found that the debris flow in this area is mainly distributed in the middle and high mountains. The start of debris flow may be caused by slides on the steep slope, debris avalanche or rockfall, or it may be caused by the spontaneous instability of the steep fluid bed^23,24^. The types of debris flow in high mountain areas can be divided into glacial type, meltwater (ice and snow) type, collapse (ice lake) type, heavy rain type and mixed type. Many scholars have carried out relevant research on the characteristics of the formation mechanism, disaster mode, hazards of these five types of debris flows^25–31^. That climate changes affect the stability of slides, debris avalanche, glacier, ice lake and have an influence on debris flows^32,33^. The research revealed that the climate changes with the change of mountain height, resulting in the formation of glacial debris flow in the upper mountain area, and the formation of heavy rain debris flow in the downstream, a mixed debris flow between the two. Few scholars associate it with the vertical mountain belt, but regard it as a natural phenomenon. According to the research of Ren Meijun^34^, Chen Guojie^16^, Guo Yongming^35^ and other scholars, the vertical mountain belt in the Upper Min River are divided into 7 zones according to the elevation: subtropical zone (< 1300 m), warm temperate zone (1301–1900 m), temperate zone (1901–3000 m), cold temperate zone (3001–3800 m), subrigid zone (3801–4400 m), frigid zone (4401–5000 m), tundra zone (> 5000 m). In fact, the development and formation of these five types of debris flow in the high mountain area are strictly related to the vertical mountain belt; the debris flows in the high mountain area can also be regarded as the product of the vertical mountain belt.

As mentioned above, the generation, formation, and development of debris flows are closely related to the vertical climate, vegetation, and soil conditions of a given mountainous area. Such a relationship between debris flows and the vertical differentiation of mountainous regions has been studied by numerous scholars. A. B. Yermakov, a former Soviet Union scholar, proposed in 1954 that the formation area of debris flows should be classified by elevation into the nival zone, the sub-nival stony rock pile zone, the alpine zone, the sub-alpine zone, and the forest zone. He further studied the types and features of loose solid materials in each zone, illustrating their influence upon the formation of debris flows^36^. Patton found that the start of debris flows in the Colorado Front Range was mainly controlled by local terrain^37^. Many scholars also clarified the correlation between the climate-vegetation-soil zones and debris flow development. Among them, Li Honglian has listed 9 regions with higher debris flow frequency in China (7 with rainstorm debris flows and 2 with the glacial)^38^. He analyzed the vegetation of these triggering zones in exploring the most favorable climate condition for debris flow development. However, he failed to notice the formation laws and hazard characteristics, not to mention the mechanism of impacts on mountainous settlements. Furthermore, there are studies on the correlation of debris flow formation with precipitation and temperature, they often separate the 2 triggers in the process of analysis; and other studies on the relationship between debris flow formation and vegetation, forest vegetation in particular, argued that debris flows tend to occur on vegetational-deteriorated mountains such as the Alps, and noticeably cease at the restoration of vegetation. However, there are still studies insisting that no direct correlation exists between vegetation variation and debris flow formation. The Reaches of Drying River in Yunnan Province serve as a frequently quoted instance with lush vegetation but intense debris flow incidence^39^. By reviewing related research, it is evident that individual factors, including climate, vegetation and soil have seldom been integrated into a study on debris flow occurrence.

There are multiple useful statistical tools that are widely recognized to compute this sort of correlation between factors and the predictive power of a variable. These methods mainly include spatial autocorrelation test Moran’s I^40^, semivariogram^41^, Ripley K^42^, hot spot detection Gi^43^, LISA^44^, SatScan^45^, multivariate Logistic regression model^46,47^; spatial regression SAR/MAR/CAR^48–50^, GWR^51^, spatial Bayesian hierarchical model BHM^52^, etc. However, these methods are mainly to analyze and use the spatial autocorrelation of data. The factors of debris flow occurrence are spatial stratified heterogeneity, so there are limitations to applying the above methods to calculate the factors influence of vertical mountain differentiation on debris flow occurrence^53^.

GEO is a set of statistical methods established by Wang Jinfeng to detect spatial differentiation and reveal the driving force behind it^54,55^. One of its significant advantages is that it can identify quantitative data as well as qualitative data. Another unique benefit of geographic detectors is to detect the interaction between two factors on the dependent variable. GEO method is proposed to solve the problem of environmental factor recognition of spatial variation of neonatal neural tube defects^54^. Thereafter GEO has been widely used to analyze the driving forces and influencing factors of various phenomena and the interaction of multiple factors. Hu et al.^55^ applied GEO to analyze the influencing factors of children’s death in the 2008 Wenchuan earthquake. Li et al.^56^ applied GEO to the analysis of factors affecting the spatial differentiation of soil antibiotics. Luo et al.^57^ applied GEO to the 8 major terrain areas in the United States to detect the leading factors of their formation. Yang et al.^58^ used GEO to explore the influencing factors and optimization strategies of the spatial distribution of rural settlements. Du et al.^59^ applied GEO to the analysis of control factors in sandy areas. Ju et al.^60^ applied GEO to the analysis of driving forces for land expansion. GEO is widely used in fields such as soil pollution, public health^61,62^, groundwater contamination, ecology^63,64^, and meteorology^65,66^. There are both qualitative data and quantitative data in the influencing factors of debris flow distribution, so GEO is very suitable for discussing the respective influence of vertical mountain differentiation on debris flow occurrence.

Taking the Upper Min River as the study area, 9 factors including vertical zones, annual average temperature, maximum daily precipitation, sunshine hours, vegetation type, soil type, slope, aspect and lithology were selected to characterize the mountain vertical differentiation. GEO model was used to explore dominant factors and interaction of 9 factors on debris flow occurrences, and to find out the high-susceptibility type or range of each factor. The study results will provide a theoretical foundation and practical reference for a better understanding of the debris flow development law, preventing and reducing damages to local people’s life and property, and promoting sustainable economic development.

## Material and methods

### Study area

**Figure 1:**
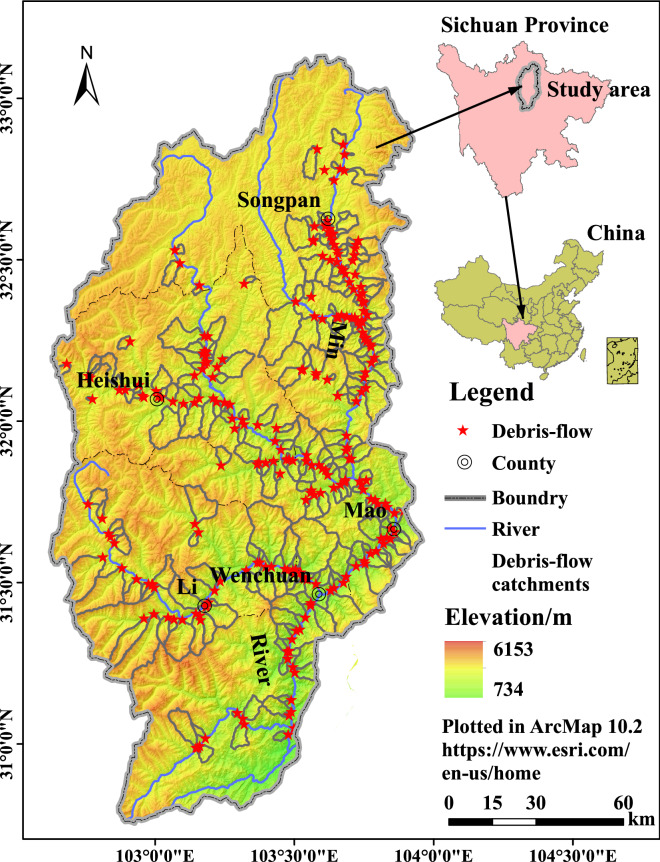
Map of the Upper Min River and debris flow distribution. The Upper Min River and debris flow extracted from ASTER GDEM V2 30 m data (https://lpdaac.usgs.gov/)^67^ and Google Earth images (Map data: Google, Maxar Technologies).

Within 31°45′N–33°09′N and 102°35′E–103°56′E, the Upper Min River refers to an area of 2.2 × 10^4^ km^2^ covered by the river and its tributaries upstream above the Dujiangyan Dam, with a north–south length of 267 km and an east–west width of 152 km, including the counties of Wenchuan, Mao, Li, Songpan and Heishui of Aba Tibetan Autonomous. (Fig. 1). Figure 1 is based on the ASTER GDEM V2 30 m data^67^, the preliminary results are extracted according to the drainage area extraction algorithm, and then generated after matching with Google Earth remote sensing images.

Located in the eastern Qinghai–Tibet Plateau, the study region serves as a transition from the hilly and mountainous area around the Sichuan Basin to the Qing–Tibet Plateau, demonstrating geomorphologic features of mountains and canyons. The Upper Min River flows from high-elevation tributaries in the northwest to its outlet in the south, where it joins the Upper Min River. The major river valleys are longitudinally south-north orientated. Steep valley sides and large longitudinal slopes have induced strong bottom and lateral erosion of the river. Due to the intensive seismic activity, among the exposed strata ranging from the Proterozoic to the Cenozoic Era, there are widely distributed continental clastic rocks, volcaniclastic rocks, carbonate rocks, and regional metamorphic rocks. Intercalated with carbonate rocks, the river valleys are concentrated by Phillies, slates, and metamorphic rocks of the Devonian (D) and Silurian (S) systems of the Paleozoic Era. The zonal arid valleys feature the study region with three-dimensional characteristics of the mountain climate. The Upper Min River belong to the alpine and plateau alpine climatic zone^16^, and , warm temperate zone, temperate zone, cold temperate zone, subrigid zone, frigid zone, ice-snow zone appear in order from the valley to the ridge. The mean annual temperature ranges from 5.7 to 13.5 °C, and the annual precipitation from 400 to 800 mm with an 80% concentration in the period from May to October. In addition to the deep river incision and large elevation disparity, the fact that warm and moist air flows have to climb over mountains before reaching this region results in scarce rainfall and dry climate, further promoting the formation of dry-hot valleys.

With a population of 384,179, the study region is the only but one largest Qiang ethnic settlement in China, and also a poverty-stricken mountainous area with comparatively limited economic development^20^. Han, Zang, Qiang, and Hui people co-inhabit in the river valleys and mountain terraces, with Wenchuan, Townships of Yingxiu and Xuankou as the densely populated center. Due to their farming culture and large population, the river valleys have gathered most of the region’s secondary and tertiary industries, while high mountains and mountain plateaus adopt animal husbandry as the main economic activity. Currently, there are 625 administrative villages with 66,500 rural households^68^ (Table 1), demonstrating settlement characteristics of large dispersion, low density, and small scale.

**Table 1:** Village distribution and economic development of the Upper Min River.

County	Land area/km^2^	Per capita GDP/Yuan	Net income per capita/Yuan	Township	Adminstrative villages	Farmer’s number	The density of adminstrative villages/km^2^	Total population
Songpan	7705	5780	1425	25	142	11,640	0.018	74,945
Heishui	3945	2656	1029	17	124	10,950	0.031	60,854
Mao	3655	3748	1222	23	152	19,219	0.042	111,452
Li	3817	6016	1112	13	81	8176	0.021	43,375
Wenchuan	3912	11262	1679	14	126	16,515	0.032	93,553
The upper Min River	23,034	5892	1293	92	625	66,500	0.027	384,179

### Environmental factors

A selection of 244 debris flows located in the Upper Min River was made from the landslide inventory provided by the Sichuan Geological Environment Management System (https://202.61.89.33:16003) under the Department of Natural Resources of Sichuan Province. The Sichuan Geological Environment Management System provides data on geological disasters (disaster types including slide, debris flow, ground fissure, ground subsidence, and ground subsidence) discovered during 1905–2018 in Sichuan Province, including 6,250 debris flows. The data is obtained through the field survey of the geological disaster investigation project organized and implemented by the Natural Resources Department of Sichuan Province every year and the residents' report, mainly including the coordinates of the geological disaster point, the specific geographic location, the time of occurrence, the threat of property, the number of threats, the number of threatened households, volume, scale and other parameters. Based on the data of 244 typical debris flow hazard points in the Upper Min River provided by the Sichuan Geological Environment Management System, the author uses ArcGIS 10.2 software to manually draw 244 debris flow catchments using the GDEMV2 30 m data^67^ and Google Earth remote sensing images in the Upper Min River as the base map (Fig. 1, Table 2).

**Table 2:** The distribution of debris flow gully in the Upper Min River.

County	Number of debris flow gully	Mainly distribution
Songpan	73	Min River: on the main stream both sides and tributaries
Heishui	44	Heishui River: on the main stream both sides and tributaries
Mao	60	Min River: on the main stream both sides and tributaries
Li	33	Zagunao River: on the main stream both sides and tributaries
Wenchuan	33	Min River: on the main stream both sides and tributaries
The upper of Min River	244	

The Upper Min River is a typical active area of debris flow in southwestern China. The debris flow is mainly concentrated in the alpine gorge area, especially on the banks of the river below Zhenjiangguan and its tributaries in the mainstream of the Upper Min River, and the Heishui River below the Heshui and its tributaries and below the Shaheba section of the Zagunao River and its tributaries. The 9 factors and the symbols used were vertical zones (D1), annual average temperature (D2), maximum daily rainfall (D3), sunshine hours (D4), vegetation type (D5), soil type (D6), slope (D7), aspect (D8) and lithology (D9). The vertical zones, slope and aspect data are extracted from the ASTER GDEM V2 30 m data^67^. The average annual temperature sunshine was obtained from the National Meteorological Center China Meteorological Data Network (https://www.escience.gov.cn/). The rainfall data comes from the Sichuan Provincial Hydrology and Water Resources Bureau. Rainfall data were collected from 79 meteorological stations in the Upper Min River during the flood season (1981–2010). The density of the rain gauge network is 3.59 × 10^–3^/km^2^. The elevation distribution of the rain gauges is from 886 m (Yingxiu) to 5286 m (Aotaiji). The soil-vegetation distribution data were obtained from Assessment Dataset of Habitat Suitability in the Upper Reaches of Min River, China^69^. The lithology data extracted from the 1: 200,000 Chinese geological map in the National geological data Museum (https://ngac.org.cn/).

First of all, ArcGIS10.2 software was used to generate 23,018 square grids of 1 km × 1 km in the Upper reaches of Min River. The 1 km × 1 km grid is selected because it is smaller than the minimum watershed area of 244 debris flows. Then the grid map is superimposed with the debris flow distribution map. The area of the debris flow in each grid is counted, and the ratio of the debris flow catchment of each grid is calculated. Then, the other debris flow impact factor layers are superimposed with the grid map to obtain relevant data and exported. Finally, GEO software is used for data analysis.

The law of vertical differentiation of mountainous areas refers to the distribution pattern of mountainous natural landscapes with elevation changes^34,35,70^. Due to the change in altitude in the vertical direction, the conditions such as water and heat are significantly different, resulting in various combinations, which in turn affect the development, development and formation of debris flow. The relative elevation difference of the Upper Min River is 1500–3000 m. The hydrothermal conditions are very different, and the vertical zonality of the climate is significant, which leads to obvious vertical zonal differences in vegetation, soil type and topography.

**Figure 2:**
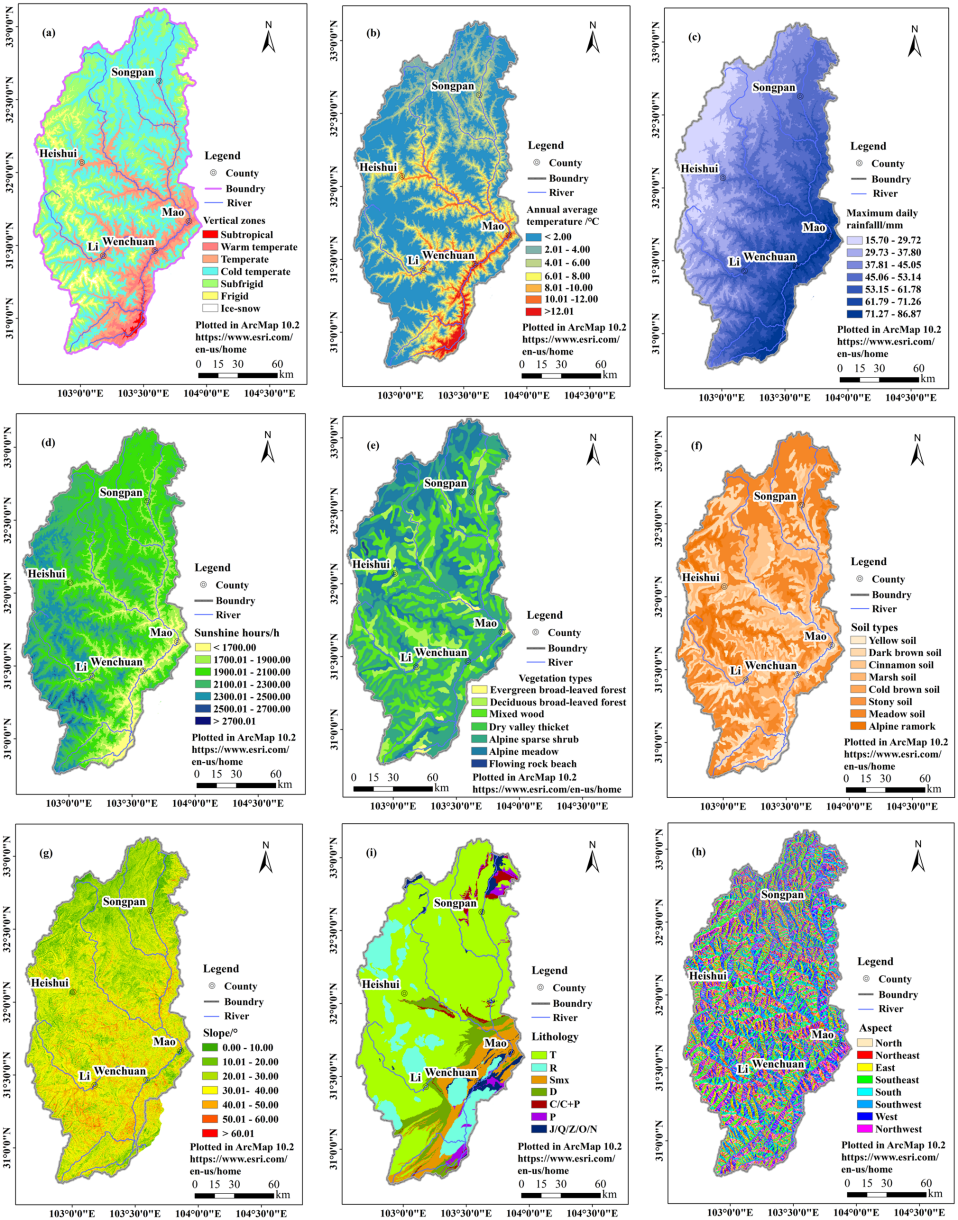
Spatial distributions of nine factors in the Upper Min River. (**a**) The vertical zones extracted from ASTER GDEM V2 30 m data (https://lpdaac.usgs.gov/)^67^. (**b**) The distribution of annual average temperature is based on the annual mean temperature data of the Upper Min River Meteorological Station. (**c**) The maximum daily rainfall data comes from the Sichuan Provincial Hydrology and Water Resources Bureau. (**d**) The distribution sunshine hours is based on the annual sunshine hours data of the Upper Min River Meteorological Station. (**e**) The distribution of vegetation extracted from Assessment Dataset of Habitat Suitability in the Upper Reaches of Min River, China (https://www.geodoi.ac.cn/WebEn/Default.aspx)^69^. (**f**) The distribution of soil extracted from Assessment Dataset of Habitat Suitability in the Upper Reaches of Min River, China (https://www.geodoi.ac.cn/WebEn/Default.aspx)^69^. (**g**) Slope extracted from ASTER GDEM V2 30 m data (https://lpdaac.usgs.gov/)^67^. (**h**) Aspect extracted from ASTER GDEM V2 30 m data (https://lpdaac.usgs.gov/)^67^. (**i**) Lithological data extracted from 1: 200,000 Chinese geological maps (https://ngac.org.cn/).

The 9 factors selected for the respective influence are as follows:

- 1. *Vertical zones (D1)* The elevation difference in the Upper Min River is huge, and the hydrothermal conditions vary significantly with elevation. The climate regions in the study area are significantly different. According to the research of Ren Meijun^39^, Chen Guojie^16^, Guo Yongming^41^ and other scholars, the Upper Min River are divided into 7 zones according to the elevation: subtropical zone (< 1300 m), warm temperate zone (1301–1900 m), temperate zone (1901–3000 m), cold temperate zone (3001–3800 m), subrigid zone (3801–4400 m), frigid zone (4401–5000 m), ice-snow zone (> 5000 m) (Fig. 2a). Figure 2a is based on the ASTER GDEM V2 30m data and is reclassified using ArcMap according to the elevation distribution of the climatic zone.
- 2. *Annual temperature (D2)* the vertical temperature of the Upper Min River is significant, which is one of the main climatic factors controlling the vertical differentiation and vegetation distribution of the mountain, and also the main factor was controlling the weathering of the rock. The development and formation of the debris flow are greatly affected. The annual average temperature is divided into 7 categories using the natural breakpoint method (Fig. 2b). Figure 2b is based on the annual average temperature data of the Upper Min River Meteorological Station using ArcMap for spatial interpolation.
- 3. *Maximum daily rainfall (D3)* The spatial distribution of precipitation caused by the undulations and complex topography of the Upper Min River. In the dry-warm valleys, precipitation is small. Generally speaking, the precipitation is mainly controlled by altitude and latitude and longitude. In the horizontal direction, the arid elliptical arid center centered on the Shaba area of Mao gradually increases the precipitation from east to west. In the vertical direction, precipitation is positively correlated with altitude, relationship, and maximum daily rainfall is divided into 7 categories using natural breakpoints (Fig. 2c). Figure 2c is based on the maximum daily rainfall data of the Upper Min River Meteorological Station using ArcMap for spatial interpolation.
- 4. *Sunshine hours (D4)* The number of sunshine hours directly affects the heat distribution in different regions, which in turn affects the climate. In general, the higher the altitude, the longer the sunshine hours, the positive correlation, showing a distinct vertical differentiation. The annual average sunshine hours were divided into 7 categories using the natural breakpoint method (Fig. 2d). Figure 2d is based on the annual sunshine hours data of the Upper Min River Meteorological Station using ArcMap for spatial interpolation.
- 5. *Vegetation type (D5)* The vertical differentiation of vegetation in the Upper Min River is obvious. From the vertical distribution of vegetation from the foothills to the top of the mountain, the main vegetation types from low to high are evergreen broad-leaved forest, deciduous broad-leaved forest, mixed wood, dry valley thicket, sparse alpine shrub, alpine meadow, flowing rocky beach (Fig. 2e). The Vegetation distribution data were obtained from Assessment Dataset of Habitat Suitability in the Upper Reaches of Min River, China^35^. Figure 2e plotted in ArcMap.
- 6. *Soil type (D6)* The soil types in the Upper Min River are diverse. Due to the vertical differentiation of climate and vegetation, the soil distribution shows a distinct vertical spectrum. The soil distribution and altitude are basically synchronized. According to its main distribution range, from low to high, it is yellow soil, dark brown soil, cinnamon soil, cold brown soil, marsh soil, stony soil, meadow soil, alpine remark (Fig. 2f). The soil distribution data were obtained from Assessment Dataset of Habitat Suitability in the Upper Reaches of Min River, China^35^. Figure 2f plotted in ArcMap.
- 7. *Slope (D7)* Slope directly affects the fluctuation of the regional landscape. The most direct manifestation is the difference in vegetation type and coverage, which in turn forms different vertical bands of the mountain. At the same time, the slope changes cause the stability of the soil and the surface hydrodynamics to change, that is, with the increase of the slope, the erosion ability of the precipitation, and the erosion intensity is also gradually increasing. The slopes in the Upper Min River are divided into 7 categories (Fig. 2g). Figure 2g is obtained from the aspect analysis of the DEM data using ArcMap.
- 8. *Aspect (D8)* Aspect is one of the important topographic factors that distinguish the vertical band spectrum of mountainous land. The influence of physiology characteristics of aspect is mainly explained by the fact that the light and heat conditions accepted on different slopes are different. Generally speaking, the solar radiation heat received by the shady slope is much smaller than that of the sunny slope, resulting in a difference in regional vegetation type distribution. According to the direction of the octant method: North, North East, East, South East, South, Nancy, West and North West (Fig. 2h). Figure 2h is obtained from the aspect analysis of the DEM data using ArcMap.
- 9. *Lithology (D9)* The lithology is divided into 7 lithological grades according to the different lithological characteristics of each stratum: Triassic marl, shale, sandy limestone (T), intrusive rocks (R) in each period, and Silurian Maoxian Group Thousands of Rocks with Crystal Limestone (Smx), Devonian Thick Layer Limestones (D), Carboniferous Crystal Limestone/Limestones with Thousand Stones (C/C + P), Permian Limestone, Gravel Rock (P), Jurassic sandstone/Quaternary clay/Sinian dolomite/Ordovician sand gravel/Neogene sand gravel (J/Q/Z/O/N). The lithological features of the stratum were converted into raster files and reclassified according to 7 lithological grades (Fig. 2i). Figure 2i plotted in ArcMap.

### Data analysis

GEO model has 4 modules^54,55^. This study mainly uses the “Factor detector”, “Interaction detector” and “Risk detector” to quantify the factors of debris flow and vertical differentiation of the Upper Min River. Analysis and discussion of the respective influence of vertical mountain differentiation on debris flow occurrence.

In this paper, factor detector is used to calculate the influence degree of the nine factors on debris flow occurrence in the Upper Min River. Factor detector is to detect to what extent a certain factor that affects the occurrence of debris flow explains the ratio of debris flow catchment. Use the explanatory power of the factor *q* value to judge, *q* value calculation formula is as follows:

1 $$ q = 1 - \frac{{\sum\limits_{h = 1}^{L} {N_{h} } \sigma_{h}^{2} }}{{N\sigma^{2} }} $$

In Eq. (1), h = 1,…, L, L are the classification of debris flow influencing factors; N_h_ and N are the grid number of the various influencing factors and the whole study area; $\sigma_{h}^{2}$ is the variance of each influencing factor; $\sigma^{2}$ is the variance of the occurrence rate of debris flow in the whole study area. The value range of *q* is [0,1]. The larger the value of *q*, the greater the influence of factors on the occurrence of debris flow, and the smaller the conversely.

Interaction detector compares the influence of two factors A and B on debris flow occurrence by interacting to detect whether different factors have interaction on debris flow occurrence so as to determine whether these two factors have a separate effect or interaction on debris flow occurrence. The evaluation method is to first calculate the q value of the two factors A and B for the ratio of debris flow catchments: q(A) and q(B), and calculate the q value when they interact: q(A ∩ B), and compare q (A), q(B) and q(A ∩ B) are compared. The relationship between the two factors can be divided into 5 categories (Table 3).

**Table 3:** GEO interaction judgment equation.

Description	Interaction
*q* (A ∩ B) > Min (*q* A), q(B))	Nonlinear antagonism
Min(*q* (A), *q* (B) < q(A ∩ B) < Max(*q* (A), *q*(B))	Single antagonist
Max(*q* (A), *q* (B)) < *q* (A ∩ B) < *q* (A) + *q*(B)	Double synergy
*q*(A ∩ B) = *q*(A) + *q*(B)	Independent
*q*(A ∩ B) > *q* (A) + *q*(B)	Nonlinear synergy

Risk detector is used to determine whether there is a significant difference between the mean values of the ratio of debris flow catchments in the two sub-regions of a certain factor, and perform a significance test. The sub-areas with greater mean significance, the more frequent the debris flow activity, used to search for areas where the debris flows frequently in each factor.

## Results

### Distribution characteristics

The distribution characteristics of debris flows in the study region are summarized as follows:

1. Concentration in deeply incised river valleys with high relief energy. The deeply incised river valleys are usually characterized by crust uplifting, tectonic activity, high relief and deep slopes, providing favorable development conditions for debris flows. Therefore, a high concentration is often witnessed in these valleys. For instance, the valleys along the Min River banks downstream below Zhenjiangguan Village of Songpan, along the Heishui River downstream below Luhua Township of Heishui, and along the Zagunao River downstream below Saba Village of Li.
2. Concentration in areas with abundant precipitation and frequent rainstorms. Intensive precipitation (rainstorms in particular) is a major trigger of mountain hazards, causing a high concentration of well-developed landslides and debris flows. The precipitation of the study region differs greatly. The period from May to September is the wet season, during which the precipitation takes up 80–85% of the total. In this period of time, mountain hazards frequently occur, proving a positive correlation with precipitation. Generally speaking, a year of abundant precipitation or climate anomalies is likely to witness mountain hazards, especially when heavy rains, rainstorms, and hailstorms are simultaneously functioning. According to statistics, the mountain hazards including debris flows, landslides, and collapses occurred during the wet season take up more than 90% of the total.

### The dominant factors of influence of vertical differentiation factors on debris flow occurrence

**Figure 3:**
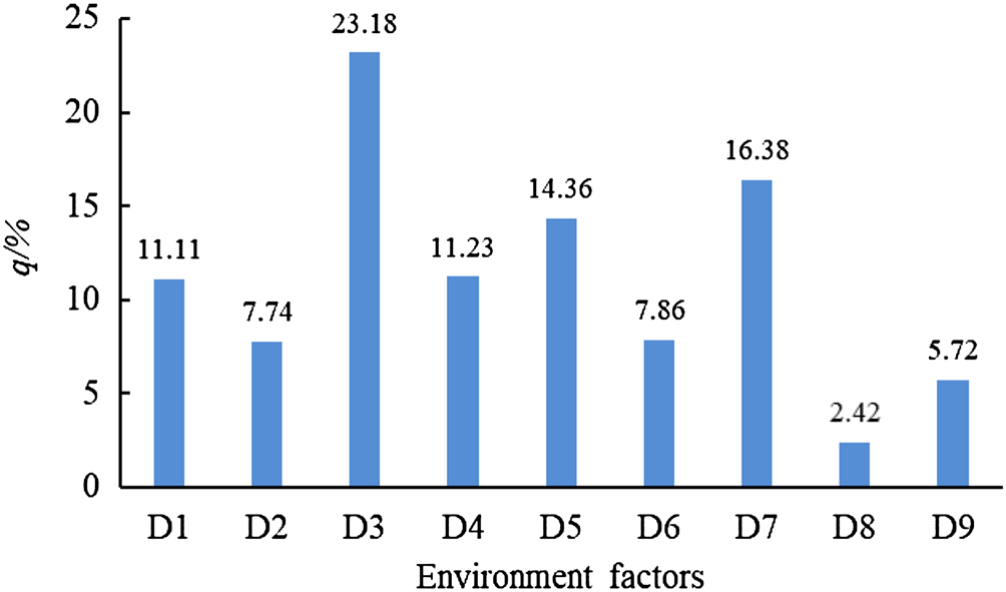
Result of risk factor detector.

The factor detector is used to measure the intensity of the influence of nine factors on debris flow occurrence. The larger the value of q, the greater the intensity of the influence of this factor on debris flow occurrence. According to the analysis results (Fig. 3), D3 (maximum daily rainfall), D7 (slope), D5 (vegetation types), D1 (sunshine hours) and D1 (vertical zones) have a influence on the debris flow of 53.92%, indicating that the occurrence of debris flow is mainly affected by the vertical differentiation of mountain, and maximum daily rainfall , slope factors in the Upper Min River has the greatest impact on debris flow occurrence in the study area, followed by the slope, vegetation types, sunshine hours and elevation.

### The interaction of vertical differentiation factors on debris flow

The interaction detector is used to test whether the two factors act independently or interact with each other. If they interact, the effect is enhanced or weakened. The interactive detector module was used to obtain the interaction of nine factors in the mountain to the debris flow (Table 4). The diagonal in Table 4 is the q value calculated by the interaction detector of 9 factors that affect debris flow occurrence. Due to local correlations and regional differences, individual factors have little effect on the occurrence of debris flows in the study area. For the comprehensive interaction of a factor with the other seven factors, the interactions with the strongest effects are: D5 ∩ D3 (vegetation type ∩ maximum daily rainfall), D3 ∩ D7 (maximum daily rainfall ∩ slope), D9 ∩ D3 (lithology ∩ maximum daily rainfall), the interaction between factors are above 5%. The interaction of nine factors in vertical mountain differentiation is nonlinearly enhanced. The effect, that is, the interaction of any two factors is greater than the independent effect of a single factor, and the additive effect of maximum daily rainfall is the most significant.

**Table 4:** Interaction of nine factors in the Upper Min River.

	D1	D2	D3	D4	D5	D6	D7	D8	D9
D1	0.02								
D2	0.03	0.01							
D3	0.05	0.04	0.02						
D4	0.02	0.03	0.04	0.02					
D5	0.04	0.04	0.06	0.05	0.02				
D6	0.03	0.03	0.05	0.03	0.04	0.01			
D7	0.05	0.05	0.06	0.05	0.05	0.05	0.03		
D8	0.02	0.02	0.03	0.02	0.03	0.02	0.04	0.00	
D9	0.04	0.03	0.06	0.03	0.04	0.03	0.04	0.02	0.01

### Risk detection of factors on debris flow occurrence

The risk detector answers the question of the geographical location of the debris flow distribution and is used to search for areas where debris flows occur frequently. In the results of the risk detector, the result information for each factor is represented in two tables. The first table gives the mean value of the area of debris flow in each zone. The second table gives a statistical difference in the mean of the attributes between every two partitions; if there is a wet difference, the corresponding value is “Y”, otherwise it is “N”.

Taking vertical zones as an example, the results of the risk detector are shown in Table 5. It can be seen from Table 5 that the elevation is divided into seven partitions, represented by the numbers 1, 2, …, 7. According to the mean value of the ratio of debris flow catchment, the elevation band is sorted, 3 > 4 > 7 > 6 > 2 > 5 > 1, and the mean value of the temperate debris flow area is the largest in vertical zone 3 (temperate zone 1901–3000 m). (Table 5).

**Table 5:** Mean value of the ratio of debris flow catchments in each vertical zone.

1	2	3	4	5	6	7
3.6014	20.9462	32.3331	27.6175	19.5501	21.8168	25.1370

Other factors can be similarly analyzed to find areas of frequent debris flow in the vertical differentiation of the mountains (Table 6). Table 6 revealed that the most frequent mountainous vertical belt of the debris flow is the temperate mixed wood stony soil.

**Table 6:** Frequent range of debris flow in various factors of mountain vertical differentiation.

Factor	D1	D2	D3	D4	D5	D6	D7	D8	D9
Partition number	3	3	7	2	3	6	5	2	3

## Discussion

According to the results of the factor detector and the risk detector, maximum daily rainfall, slope factors are the main spatial drivers of debris flow. Maximum daily rainfall in the main risk area of debris flow is 71–86 mm, which happens to be in the intense rainstorm area in the middle and eastern part of the Upper Min River. Among them, arid and dry valleys with the mainstream of the Min River in Zhenjiangguan of Songpan County and Mianzhu Town of Wenchuan County are typical. Although its annual precipitation is very small, concentrated rainfall during the flood season is prone to induce debris flow. The slope of the main risk area for debris flow is 5 (40°–50°). With the increase of the slope, the scouring ability and erosion intensity of rainfall also gradually increase. The great height difference makes the loose material sources such as slop deposits have higher potential energy conditions. At the same time, the slope of the gully has more restrictive effects on the movement speed of the debris flow, runoff conditions and deposition. Therefore, the debris flow is concentrated in deeply incised river valleys with high relief energy.

**Figure 4:**
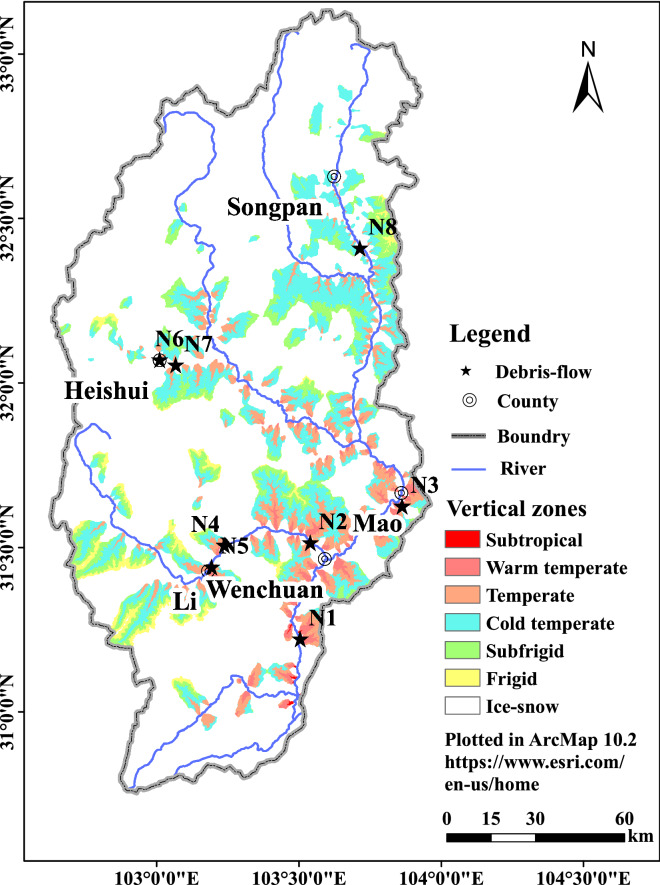
The vertical zones of 244 debris flow gullies in the Upper Min River. The map extracted from ASTER GDEM V2 30 m data. (https://lpdaac.usgs.gov/)^67^.

Being ecologically fragile within Upper Yangtze and prone to mountain hazards, the study area demonstrates distinct characteristics of mountain vertical zonality (Fig. 4). Figure 4 is obtained by extracting climate zone grids from 244 debris flow gully basins in ArcMap. An obvious respective influence of vertical mountain differentiation on debris flow occurrence is witnessed. The debris flow accumulation area and propagation area on the Upper Min River are mainly distributed on the banks of the Min River and its tributary valleys at an altitude of Wenchuan (1300 m)–Songpan (2800 m), located in the subtropical and warm temperate arid valleys. With limited annual precipitation of 400–800 mm, the subtropical and warm temperate arid valleys provide favorable conditions for the debris flow occurrence. Intensive and concentrated precipitation brought by storms may induce a simultaneous burst of several or even a dozen debris flows around the rainstorm center. The debris flow formation and clear-water confluence areas usually cover one or two base zones: a debris flow deposed in the subtropical zone will have its formation and clear-water confluence areas in the warm temperate and temperate zones; while that deposed in the warm temperate zone will often have its formation and clear-water confluence areas in the temperate and cold temperate zones (Fig. 4).

**Figure 5:**
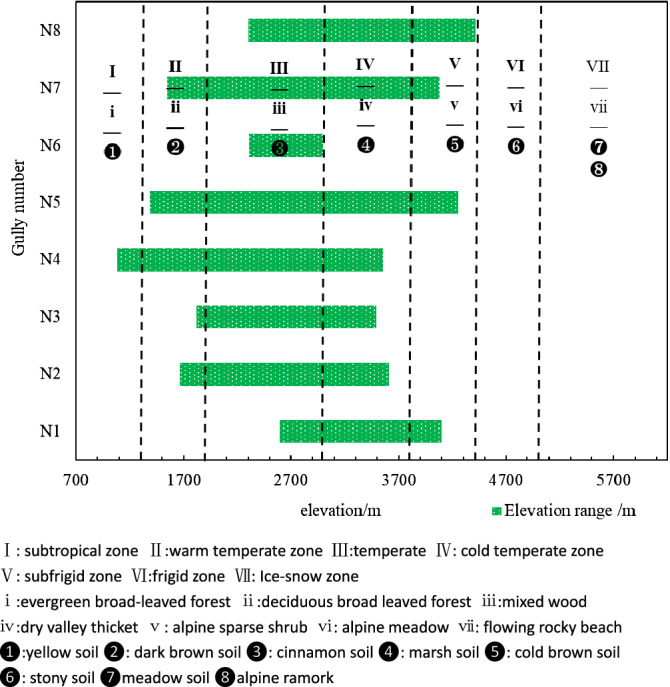
Relationship between the debris flow gullies and mountain vertical zones in the study region.

The uneven mountain vertical differentiation across climate-vegetation-soil zones has exerted a deep influence on the debris flow occurrence: a debris flow gully usually has an elevation above 1000 m. Among all the selected gullies (including Fotangba gully (N1) and Chayuan gully (N2) in Wenchuan, Longdong gully (N3) in Mao, Haermu gully (N4) and Ridiwa gully (N5) in Li, Luhua gully (N6) and Luobagully (N7) in Heishui, and Longtanbao gully (N8) in Songpan), Luhua gully (690 m) is the only one below 1000 m (Figs. 4, 5). The rest gullies accordingly have crossed more than one climate-vegetation-soil zones.

On the one hand, the deposition and propagation areas of the above-mentioned gullies are all located within base zones (within the base zone transiting from the subtropical zone to the warm temperate zone: Fotangba gully; within the warm temperate base zone: Tea garden gully, Longdong gully, Haermu gully, and Ridi Village gully; within the temperate base zone: Luhua gully, Luoba Street gully, and Longtanbao gully). On the other hand, the formation and clear-water confluence areas are often situated in the warm temperate and temperate zones (Fotangba gully), in the temperate zone (Tea garden gully, Longdong gully, Haermu gully, and Ridi Village gully), and in the cold temperate and temperate zones (Longtanbao gully).

## Conclusions

In this study, the Upper Min River was used as the study area, and vertical zones, annual average temperature, maximum daily rainfall, sunshine hours, vegetation type, soil type, slope, aspect and lithology 9 factors representing the vertical differentiation characteristics of the mountain were selected. The model quantitatively analyzes the relationship between the debris flow and the nine factors that characterize the vertical zone of the mountain and conducts the respective influence of vertical mountain differentiation on debris flow occurrence. It will expand the understanding of the development law of the debris flow disaster, and also determine the debris flow disaster on the Upper Min River. The research provides guidance and provides a richer scientific basis for disaster prevention and mitigation management of debris flow disasters. This study mainly got the following three conclusions:

1. Debris flows of the study region are mainly distributed along the Min River and its tributaries downstream below Zhenjiangguan, along the Heishui River and its tributaries downstream below Heishui County, along the Zagunao River and its tributaries downstream below Shaba. These valleys are also prone to rainstorms and landslides, due to deep incision, high relief, and abundant precipitation.
2. Our results reveal that maximum daily rainfall, slope factors are the main spatial drivers of vertical mountain differentiation on debris flow occurrence. The factor detector reveals the consistency and difference of the P_D, H_ interpretation of the influence factor on the debris flow. The spatial data analysis of debris flow indicates that the debris flow in the Upper Min River has vertical differentiation. The main vertical distribution factors of the main mountain with influence above 10% are maximum daily rainfall (23.18%), slope (16.38%), vegetation type (14.36%), sunshine hours (11.23%), elevation (11.11%). The interaction detector proves that the spatial distribution of debris flow is the result of multiple factors of vertical differentiation of mountains, and the interaction between different factors is different, with significant differences. The results show that the interaction between the vertical differentiation of the mountainous areas in the study area is a nonlinear enhancement, that is, the interaction of any two factors is greater than the independent effect of a single factor, and the cumulative effect of maximum daily rainfall is the most significant.
3. The debris flow accumulation area and propagation area of the Upper Min River are mainly distributed in the arid valleys of subtropical and warm temperate zones, and the formation and clear-water confluence areas across one or two base zones. The most frequent mountainous vertical belt of debris flow is temperate coniferous and broad-leaved forest stony soil.

According to such results, the ethnic zonality also shows characteristics of vertical differentiation in the study region, where ethnic mountain settlements are concentrated and the livelihood means is highly consistent with mountain vertical zone^71^. With inherent fragility and high risk, these mountain settlements are the most-seriously-impacted geographic unit in terms of damages to livelihood resources, residents’ life and property. They are also major bearers for mountain hazards like debris flows. The interaction between mountain settlements and debris flow activities has a direct correlation with the vertical mountain differentiation. In this regard, close attention should be paid to such interaction of the two and their combined effect, combining mountain settlements, debris flows, and vertical mountain differentiation into an integrated system. Accordingly, the coupling mechanism of settlement hazards and vertical mountain differentiation has been established, providing the study region with decision-making reference for population readjustment, mountain settlement plan and site-selection.
