# Author Correction: Respective influence of vertical mountain differentiation on debris flow occurrence in the Upper Min River, China

**DOI:** 10.1038/s41598-020-73766-x

**Published:** 2020-10-08

**Authors:** Mingtao Ding, Tao Huang, Hao Zheng, Guohui Yang

**Affiliations:** Faculty of Geosciences and Environmental Engineering, Southwest Jiaotong University, Chengdu, 611756 China

Correction to: *Scientific Reports* 10.1038/s41598-020-68590-2, published online 16 July 2020.

In Figure 1, the legend for ‘Debris-flow catchments’ is missing. The correct Figure 1 appears below.

**Figure 1:**
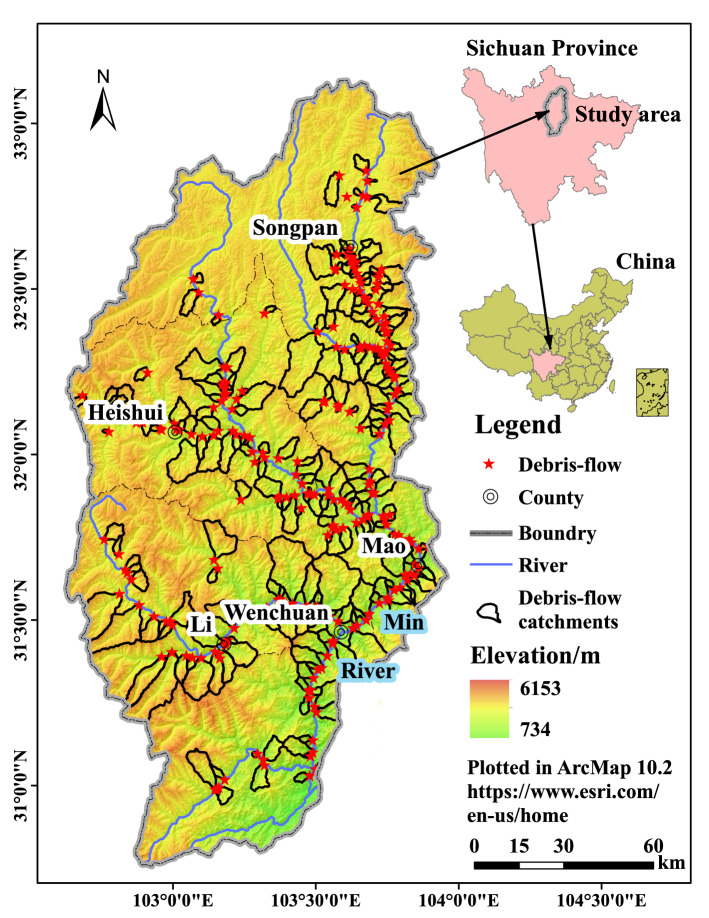
Map of the Upper Min River and debris flow distribution. The Upper Min River and debris flow extracted from ASTER GDEM V2 30 m data (https://lpdaac.usgs.gov/)^67^ and Google Earth images (Map data: Google, Maxar Technologies).

